# *Mycobacterium fortuitum* abdominal wall abscesses following liposuction

**DOI:** 10.4103/0970-0358.41113

**Published:** 2008

**Authors:** Hussam Al Soub, Eman Al-Maslamani, Mona Al-Maslamani

**Affiliations:** Department of Internal Medicine, Hamad Medical Corporation, Doha-Qatar; 1 Department of Pediatrics, Hamad Medical Corporation, Doha-Qatar

**Keywords:** Atypical mycobacteria, liposuction, *Mycobacterium fortuitum*

## Abstract

We describe here a case of abdominal abscesses due to *Mycobacterium fortuitum* following liposuction. The abscesses developed three months after the procedure and diagnosis was delayed for five months. The clues for diagnosis were persistent pus discharge in spite of broad spectrum antibiotics and failure to grow any organisms on routine culture. This condition has been rarely reported; however, the increasing number of liposuction procedures done and awareness among physicians will probably result in the identification of more cases. Combination antibiotic therapy with surgical drainage in more extensive diseases is essential for cure.

## INTRODUCTION

Cosmetic surgery including face-lift, augmentation or reduction mammoplasty and abdominoplasty with skin and fat resection are the some of the most common procedures performed in public and private hospitals.[1] More recently, suction of subcutaneous fat (liposuction) and injection of homologous fat tissue (liposculpture) have also become frequent surgical procedures.[2] Approximately 150,000 liposuction procedures were performed in the USA in 1997 and the number of procedures is increasing dramatically.[3] As a non-contaminated elective surgery, infections following liposuction are rare[4] but significant considering the large number of procedures done. Recovered microorganisms usually include common skin and soft tissue pathogens such as *Staphylococcus aureus*.[5] Nevertheless, more fastidious organisms such as *Mycobacterium fortuitum* and *Mycobacterium chelonae* are occasionally involved.[6] Most nosocomial infections with these rapidly growing mycobacteria occur sporadically, however outbreaks have also been reported.[6] Most of these infections are believed to be secondary to breaches in infection control measures related to the procedure such as the use of contaminated tap water while cleaning the cannulae used in liposuction and inadequate sterilization of surgical equipment by autoclaving.[7] We herein report a case of multiple abdominal wall abscesses caused by *Mycobacterium fortuitum* following liposuction in an attempt to increase awareness of physicians to this uncommon but serious infectious complication.

## CASE HISTORY

**Figure 1:**
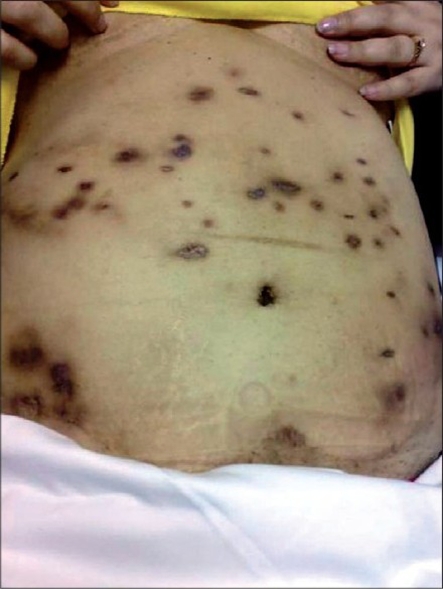
Photograph of the abdomen of the patient showing multiple scars, sinuses and large abscesses

A 47 year-old Qatari female patient was admitted to Rhumaileh hospital on 24^th^ April 2006, for elective abdominal liposuction which was done on the following day. Her past history was unremarkable except for dermolipectomy and breast reduction surgery in 2000 and diabetes mellitus for which she was maintained on oral hypoglycemic agents. She received intravenous Ceftriaxone perioperatively and for a total of three days. Her postoperative course was uneventful and she was discharged on the 3^rd^ post-operative day. She was readmitted 3 months later with signs of having developed multiple abdominal wall abscesses. Incision and drainage of abscesses was done. Examination of drained pus revealed moderate pus cells, no organisms on gram stain and culture was negative. During her stay in the hospital, she was treated with intravenous Ceftriaxone which was continued for a total of ten days. No antibiotics were given on discharge. She was readmitted again 2 weeks later with recurrence of the abdominal wall abscesses. Incision and drainage was again done. Bacterial culture of drained pus was once again negative. Mycobacterial infection was not considered so culture was not done. During this period of hospitalization, she was given intravenous Cloxacillin for 12 days and was discharged home on oral Ciprofloxacin 500 mg twice daily and Amoxycillin/Clavulinate 625 mg thrice daily. In spite of this treatment, a third incision and drainage was again necessary 1 week after her discharge. Because of these repeated recurrences in spite of prolonged treatment, she was finally referred to the Infectious Diseases Clinic 6 weeks later. Her physical examination at this time was unremarkable except for multiple abdominal wall scars, sinuses draining pus and multiple nonruptured abscesses distributed all over her abdomen [Figure 1]. The abscesses were very tender. Aspiration of one of the abscesses yielded 20 ml of thick pus. Gram stain of the pus revealed profuse pus cells but no organisms. Routine bacterial and fungal cultures of the pus were negative. She was given oral Moxifloxacin 400 mg daily for two weeks but with no response. Repeated aspiration of abscesses was done and pus was sent for bacterial culture as well as for acid-fast stain and mycobacterial culture. Results of bacterial culture and acid-fast stain were negative. By now there was a clinical suspicion of infection with atypical mycobacteria. She was therefore treated empirically with Doxycycline and Clarithromycin to which she did not respond either. Later on, the results of mycobacterial culture came positive for *Mycobacterium fortuitum* sensitive to Ciprofloxacin, Amikacin and Linezolid. The patient was however unwilling to take further antibiotics and decided to go to USA for treatment. In USA, culture of the pus grew the same organism with the same sensitivity. She underwent a course of Linezolid and Ciprofloxacin for four months with complete healing of abscesses.

## DISCUSSION

*Mycobacterium fortuitum* is one of several rapidly growing mycobacteria, which are increasingly being recognized to cause human disease.[8] Rapidly growing mycobacteria are found ubiquitously in the environment including water, soil, dust, wild and domestic animals and fish.[8] They can grow in municipal water systems and in distilled water and are resistant to sterilizers, antiseptics and other standard disinfectants including 10% povidine-iodine, 2% aqueous formaldehyde and 2% alkaline glutaraldehyde.[9] These mycobacteria are known to cause cutaneous infections, typically in association with trauma or clinical procedures. Nosocomial disease outbreaks have been reported and include surgical wound infections, post-injection abscesses and bone infections.[10]

The characteristic features of surgical wound infections caused by *M. fortuitum* resemble that of pyogenic abscesses and include local erythema, induration, microabscesses and discharge from sinuses. Fever, chills and systemic manifestation are infrequent. The median time of onset of symptoms after surgery is five weeks (range 1-20 weeks). Spontaneous recovery is rare.[2] In a series of 63 patients with skin and soft infections due to rapidly growing mycobacteria reported by Uslam *et al.*,[11] patients infected with *M. fortuitum* were found to be younger and less likely to be immunocompromised than patients infected with *M. chelonae.* More patients with *M. fortuitum* had a prior invasive surgical procedure at the infected site. They also found that diagnosis is frequently delayed as mycobacterial cultures are not routinely performed. The median time from onset of symptoms and the microbiologic diagnosis was 86 days.[11]

Treatment of infections caused by *M. fortuitum* depends on both the antimicrobial susceptibilities of the isolate and the clinical manifestation. No controlled studies have established the optimal therapeutic regimen. In general, the organism is usually susceptible *in vitro* to amikacin, cefoxitin, imipenem, sulfonamides and flouroquinolones.[2,13] It must be emphasized that skin and soft tissue infections caused by *M. fortuitum* can be treated successfully only through a combined approach - with surgical removal of all necrotic, drainage of purulent accumulations and antibiotic therapy. Wounds must be left open and packed to prevent early closure of skin, which can result in the reaccumulation of pus and the appearance of new draining fistulas.[2] Combination therapy seems prudent initially because of concerns about resistance.[11,14] It should include two or three antibiotics to which the organism is susceptible. Treatment should be continued for three months or until the resolution of clinical manifestations.

Liposuction is an increasingly performed cosmetic procedure. The number of liposuction procedures performed by members of the American society for Aesthetic Plastic Surgery (ASAPS) more than tripled between 1992 and 1997 and there was an additional 50% increase between 1997 and 1999. Many more procedures are performed by non-ASAPS members.[3] Interestingly, in spite of the large number of liposuction procedures performed, post liposuction infections with rapidly growing mycobacteria are rare. The first official report to the Centers for Disease Control and Prevention of an outbreak of surgical site infection (SSI) due to *M. fortuitum* following liposuction was made in 1998.[12] In that report, two out of seven culture-confirmed cases of SSI following liposuction or liposculpture were caused by *M. fortuitum*. Following that report, there have been four more reports of rapidly growing mycobacterial skin and soft tissues infections following liposuction. Of these, only one was due to *M. fortuitum.*[13] This finding indicates that post liposuction infection with *M. fortuitum* is quite rare. However under-reporting is a likely contributing factor as infections with non tuberculous mycobacteria are not required to be reported in many countries. Approximately 300 liposuction procedures were performed in 2006 in Qatar; however, to our knowledge, this is the first case of post liposuction infection with *M. fortuitum* seen in Qatar.

The presentation in our case was very similar to that reported by others with multiple abdominal wall abscesses developing three months after liposuction. It conforms very much to cases of *M. fortuitum* skin and soft tissue infections reported by Uslam *et al*.[11] in that our patient was also relatively young, immunocompetent and had had a prior invasive surgical procedure.

Several methods are used to sterilize instruments in liposuction. Quaternary ammonium compounds were frequently used in the past; however these compounds are considered low-level disinfectants that cannot destroy mycobacteria. They were incriminated in the outbreak described previously.[12] Following that outbreak, the Centers for Disease Control and Prevention recommended the use of high-level disinfection using 2% glutaraldehyde, ethylene oxide gas sterilization or steam autoclaving to sterilize equipments used in liposuction.[12] One or two liposuction procedures are performed in our hospital on each working day. Sterilization of the equipments used is done by autoclave using steam under pressure. We believe that mycobacteria were introduced in our patient at the time of surgery through contaminated cannulae.

Our case demonstrates the difficulties in making the diagnosis of infections with rapidly growing mycobacteria. Diagnosis was delayed in our patient for almost five months. Delayed diagnosis of cutaneous infections due to *M. fortuitum* is a common problem. Causes of delayed diagnosis are multiple and include resemblance to pyogenic infections and lack of awareness of the condition or a low index of suspicion among physicians. The fact that these organisms are highly susceptible to NaOH used to remove other bacteria from specimens and they do not always stain with Zeihl-Neelsen or Kinyoun adds to the difficulty in identifying these organism thus leading to delayed diagnosis.[14] Culture is the gold standard for diagnosis of these infections. Keys for early diagnosis include keeping a high index of suspicion, performing Zeihl-Neelsen or Kinyoun stains on all tissues obtained during debridement of infected wounds following liposuction and alerting the microbiology laboratory to the possibility of infection with atypical mycobacteria.

In conclusion, cutaneous infections with *M. fortuitum* following liposuction are very rare. Meticulous attention to infection control and proper sterilization of equipments used during the procedure are essential for prevention. Diagnosis is frequently delayed. Keeping a high index of suspicion is essential for diagnosis. Clues for diagnosis include a chronic cutaneous infection presenting with pus-discharging nodules with no response to the usual antibiotic therapy. Treatment requires prolonged antibiotic therapy guided by susceptibility testing. Surgery is an important adjunct tool in the treatment of these infections.
